# Hepatothorax: A Rare Outcome of High-Speed Trauma

**DOI:** 10.1155/2011/905641

**Published:** 2011-11-24

**Authors:** Matthew Porcelli, Oksana Prychyna, Andrew Rosenthal, Joseph DeCostanza

**Affiliations:** Division of Trauma Services, Memorial Regional Hospital, 3501 Johnson Street, Hollywood, FL 33021, USA

## Abstract

Diaphragmatic ruptures are the result of severe blunt trauma or penetrating trauma. Motor vehicle crashes are a common mechanism associated with blunt diaphragmatic rupture (BDR). Incorporating diagnostic tools and laparotomy assist in the diagnosis and treatment of BDR. However, diagnosing BDR can be a challenge for practitioners. Early diagnosis and treatment improve the patient's outcomes. This paper details the events of a patient received in a level I trauma unit.

## 1. Introduction

Diaphragmatic rupture as a result of blunt trauma is an unusual entity usually seen in the setting of high-speed motor vehicle collisions. The low incidence and difficultly in diagnosis of blunt diaphragmatic rupture (BDR) can result in delayed treatment. Morbidity rate and mortality rate for this type of injury increase when the diagnosis is delayed. From a surgical standpoint, the diaphragm is more likely to contract and fibrose when diagnosis is delayed which makes the repair more challenging. When the diagnosis is missed completely, the patient risks herniation of abdominal contents and possible strangulation of the bowel. The threat of herniation is always present due to constant negative intrathoracic pressure and can occur as late as 50 years after the initial injury [1].

## 2. Case Report

**Figure 1:**
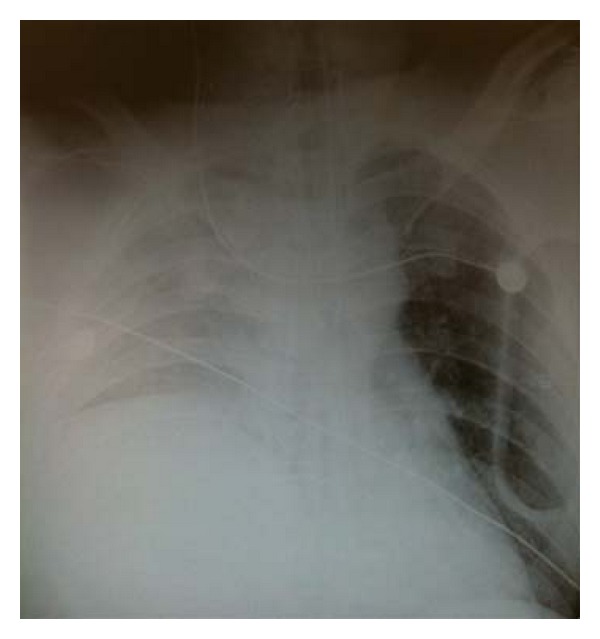
Chest X-ray on arrival to trauma bay.

A 46- year- old male was brought to our level 1 trauma center following a high-speed motorcycle collision. On arrival, the patient was unresponsive and in respiratory distress. He was immediately intubated and a chest X-ray was obtained (see Figure 1). The initial radiograph revealed that the right hemidiaphragm was elevated. The patient's arterial blood gas on admission to the trauma bay was PH 7.17, PCO2 42 mmHg, PO2 111 mmHg, HCO3 15 mmol/L, and TCO2 17 mmol/L. The patient's heart rate maintained sinus tachycardia at a rate of 130 beats per minute (BPM) to 140 BPM, systolic blood pressure fluctuated between 80 mmHg and 60 mmHg, and diastolic blood pressure fluctuated between 40 mmHg and 45 mmHg. The pelvis was grossly unstable and a commercial binder was placed to close the pelvic ring. Focused assessment sonography in trauma (FAST) scan was performed and suggested right retroperitoneal hematoma and a poorly visualized right diaphragm. There were no signs of bleeding at the meatus. A Foley catheter was inserted and placement revealed gross hematuria. Computed tomography of the chest and abdomen was deferred in order to transfer the patient expeditiously to the operating room.

The patient was brought immediately to the operating room. Exploratory laparotomy was performed and confirmed right posterior lateral diaphragmatic rupture as previously suggested on the chest radiograph. The diaphragmatic laceration extended from lateral chest wall to the pericardium (20–25 cm in length), with the majority of the liver herniating into the thorax. In addition to this injury, laparotomy revealed multiple lacerations to the transverse colon and liver and extensive contusions to the bladder wall.

**Figure 2:**
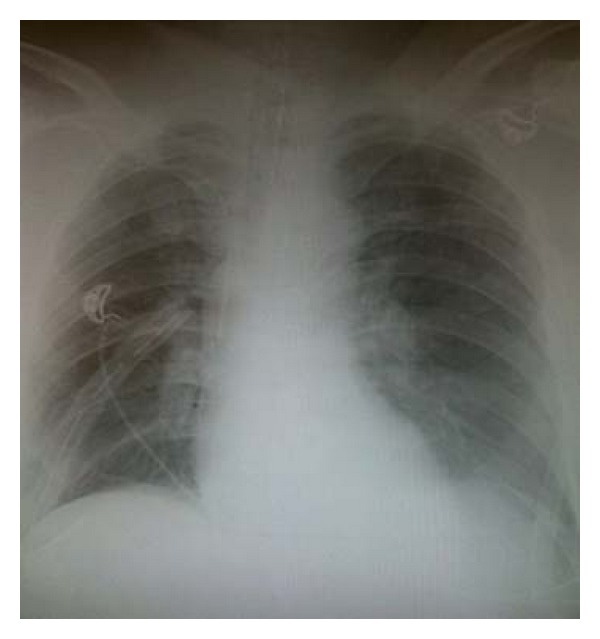
X-ray depicting repair of diaphragm.

The patient underwent diaphragm repair and damage control laparotomy. The diaphragm laceration was repaired with O-Ethibond interrupted suture placed in horizontal mattress fashion, and a second layer of the same technique was repeated for a tension-free, complete repair of the diaphragm (see Figure 2). A right chest tube was placed. The abdomen was packed and left open; a subsequent staged delayed closure was planned over the ensuing week. His pelvic fracture was treated with external fixation. Following the operation, computed tomography scan (CT) revealed a subarachnoid hemorrhage, C2 vertebral body fracture, T9 burst fracture, open-book pelvic disruption, and retroperitoneal hematoma. His subsequent hospital course was smooth, and he was discharged to a rehab facility 4 weeks after the initial injury. He is currently doing well 6 months after injury and has had no pulmonary or abdominal complications.

## 3. Discussion

Diaphragmatic rupture is a rare but life-threatening event occurring in 0.8–1.6% of patients sustaining blunt trauma [2–5]. A tremendous amount of intra-abdominal pressure is required to exceed the strength of the diaphragm muscular and tendinous composition, which explains why only a few mechanisms can generate the necessary force. High-speed motor vehicle accidents account for 90% of all BDR; therefore, other serious injuries are associated with BDR. Associated injures are seen in as many as 80–100% of BDR cases, the most common being hemothorax or pneumothorax, and injuries to the spleen or liver [6, 7].

The occurrence of left-sided BDR is nearly 3 times more common than that is right-sided BDR [8]. The discrepancy is usually attributed to the liver cushioning the impact on the right side. A more forceful and traumatic impact is required to exceed the protective properties of the liver. This increased impact could explain why patients with right-sided ruptures often have a lower Glascow coma score (GCS), higher incidence of shock, and higher overall mortality [3, 9]. Several authors have argued that the higher mortality rates seen with right-sided ruptures prevent many cases from being reported and, if autopsy discoveries were to be included, the proportions would actually be equal [3, 10, 11].

To date there is no gold standard for early and reliable diagnosis of diaphragmatic rupture. It has been estimated in previous reports that chest radiographs are diagnostic in as few as 1 out of every 3 patients and that sensitivity worsens if the patient is intubated [12, 13]. Although FAST scans are used primarily to detect free intraperitoneal blood, they can occasionally detect a diaphragmatic defect. CT scans increase the chances of preoperative diagnoses. However, in one of the largest retrospective BDR studies to date, CT scans confirmed the diagnosis in only 6% of patients [8].

The sensitivity of preoperative diagnosis is greatest when herniation of abdominal contents is obvious on chest X-ray. However, it becomes limited in the presence of hemothorax or if the rupture occurs on the right side. Right-sided BDR is not as easy to detect with imaging because the liver often obscures the diaphragm borders. [12, 14]. This pitfall in diagnosis also contributes to the delay in treatment; subsequently higher mortality rates are associated with right-sided BDR. If the patient is stable and does not have other contraindications for laparotomy, a laparoscopy can serve as an effective and less invasive means to inspect the diaphragm. Laparoscopy also has a therapeutic potential as experimental data has demonstrated successful corrective rates equal to that of open repair for closure of small defects (2 cm) [15]. Due to the high incidence of associated injuries with BDR, the diagnosis is often made during exploratory laparoscopy. Although quite helpful in detecting previously undiagnosed diaphragm injuries, laparotomy still misses the defect up to 15% of the time [6]. As the rupture is relatively easy to appreciate when sought out, it is presumably the lack of suspicion and focus on the more obvious and common injuries that leads to the missed diagnosis.

Patient presentation can vary widely depending on the degree of diaphragmatic injury. Pathognomonic signs and symptoms are often lacking or are overshadowed by associated critical injuries. The diagnosis remains challenging with preoperative success rates varying considerably, ranging from 30% to 61% [12, 16, 17].

It is possible that the trauma centers that excel in preoperative diagnosis are those that have more experience with the injury (level 1 trauma centers) and maintain a greater level of clinical suspicion. These centers tend to order repeat chest radiographs within 6 hours, a measure shown to increase diagnostic sensitivity [12, 18, 19]. It is also reasonable to assume that a high clinical suspicion preoperatively will translate into a greater intraoperative suspicion and thus greater overall diagnostic rates.

One imaging modality that could potentially increase the chances of preoperative diagnoses is the sonography. The FAST scan is routinely performed in patients sustaining blunt trauma. In several studies, the diagnosis of diaphragmatic rupture was facilitated by this technique [5, 20–22]. The FAST scan has been reported to visually detect immobility of the hemidiaphragm, a strong marker of diaphragmatic rupture in a blunt trauma patient. In addition, when set to m-mode, the FAST ultrasound reveals no excursion with respiratory effort in patients with diaphragmatic rupture [20]. Another application of sonography is screening for “rip's absent organ sign,” an indirect marker for diaphragmatic herniation. When abdominal contents herniate through the diaphragm, they tend to lie anterior to the spleen and heart. Air-filled hollow organ blocks the penetration of ultrasound waves therefore rendering the heart and spleen difficult to visualize [5]. Under the circumstances of blunt trauma, these findings can indicate diaphragmatic injury and warrant further attention.

## 4. Conclusion

BDR remains a rare and difficult diagnostic finding. Maintaining a high index of suspicion gives the greatest chance of diagnosis. Diagnosing BDR when the injury presents allows for a prompt repair and avoids strangulated bowel occurring after a delayed herniation. Maintaining high suspicion requires a tailored utilization of routine trauma imaging modalities. Repeating chest radiographs in blunt trauma patients increases diagnostic sensitivity. Also, under the circumstances of blunt trauma, adding a scan of the diaphragm to the FAST exam can raise suspicion for injury and aid in making a diagnosis.
